# Prenatal Ultrasound Detection and Neonatal Nasogastric Tube Feeding Follow Opposite Gradients Across Orofacial Cleft Phenotypes: A Nationally Ascertained Referral-Centre Cohort Study

**DOI:** 10.3390/children13091124

**Published:** 2026-08-22

**Authors:** Antonia Tarle, Marko Tarle, Marina Raguž, Sanda Huljev Frković, Predrag Knežević

**Affiliations:** 1 School of Dental Medicine, University of Zagreb, 10000 Zagreb, Croatia; mtarle@kbd.hr (M.T.); pknezev@kbd.hr (P.K.); 2 Department of Maxillofacial and Oral Surgery, Dubrava University Hospital, 10000 Zagreb, Croatia; 3 Department of Neurosurgery, Dubrava University Hospital, 10000 Zagreb, Croatia; maraguz@kbd.hr; 4 School of Medicine, Catholic University of Croatia, 10000 Zagreb, Croatia; 5 Department of Paediatrics, University Hospital Centre Zagreb, 10000 Zagreb, Croatia; shuljev@kbc-zagreb.hr; 6 School of Medicine, University of Zagreb, 10000 Zagreb, Croatia

**Keywords:** orofacial cleft, cleft palate, prenatal diagnosis, ultrasound screening, feeding difficulties, neonatal care

## Abstract

**Highlights:**

**What are the main findings?**

- Prenatal detectability and neonatal nasogastric tube feeding followed opposite gradients across orofacial cleft phenotypes, with cleft palate only showing the lowest prenatal detection (2.0%) but the highest tube-feeding requirement (24.8%).
- Among infants requiring nasogastric tube feeding, 93.5% had not been identified prenatally, revealing a clinically important mismatch between antenatal detectability and early neonatal support needs.

**What is the implication of the main finding?**

- Prenatal diagnosis alone cannot reliably identify newborns who will require early feeding support, particularly those with palatal clefts.
- Structured neonatal feeding assessment should therefore be considered for every newborn with a palatal cleft, irrespective of prenatal finding.

**Abstract:**

Background/Objectives: Cleft palate is developmentally and clinically distinct from cleft lip with or without cleft palate. Prenatal ultrasound reliably identifies clefts involving the lip but performs poorly for the secondary palate, whereas early feeding difficulty is concentrated in palatal clefts. These two observations have not previously been quantified within a single cohort. We aimed to determine whether prenatal detectability and neonatal feeding support requirements follow opposite gradients across cleft phenotypes. Methods: We conducted an ambispective study of a nationally ascertained referral-centre cohort of 328 consecutive children with an orofacial cleft and no clinically recognised syndrome, managed at the national referral centre for cleft surgery in Croatia. The cohort comprises children reaching surgical care and is not a population-based birth registry. Documented prenatal ultrasound diagnosis and feeding method during the first weeks of life were abstracted from medical records; cleft phenotype was confirmed by clinical examination. Proportions were compared with chi-square tests and exact binomial confidence intervals, and factors independently associated with each outcome were identified by multivariable logistic regression. Results: Overall prenatal detection was 76/316 (24.1%; 95% CI 19.4–29.2). Detection differed markedly by phenotype: 38.2% for cleft lip only, 34.0% for cleft lip and palate and 2.0% for cleft palate only (*p* < 0.001). Nasogastric tube feeding was required by 46/325 children (14.2%; 95% CI 10.6–18.4) and followed the opposite gradient: 0.0%, 14.2% and 24.8%, respectively (*p* < 0.001). Isolated cleft palate showed the strongest independent negative association with prenatal detection (adjusted OR 0.037; 95% CI 0.009–0.154) and was independently associated with tube feeding (adjusted OR 3.07; 95% CI 1.37–6.89). Of children requiring tube feeding, 43/46 (93.5%; 95% CI 82.1–98.6) had not been detected prenatally. Observed detection rose with year of birth in unadjusted analysis (OR 1.095 per year; 95% CI 1.012–1.185; *p* = 0.023) but was attenuated and no longer significant after adjustment (OR 1.071; 95% CI 0.991–1.157; *p* = 0.082); the temporal trend is therefore reported descriptively. An initial refer result at newborn hearing screening was markedly more frequent in children with palatal involvement than in those with cleft lip only (2.8%, 31.6% and 25.5% for cleft lip only, cleft lip and palate and cleft palate only; *p* < 0.001), contrasting cleft lip only with clefts involving the palate rather than following a monotonic gradient. Conclusions: Prenatal detectability and neonatal nasogastric tube use follow opposite gradients across cleft phenotypes. Children with cleft palate only are simultaneously the least likely to be identified before birth and the most likely to require assisted feeding, and almost all tube-fed infants arrive without a prenatal diagnosis. These findings support, as a practice consideration, routine structured neonatal feeding assessment for every newborn with a palatal cleft irrespective of prenatal findings.

## 1. Introduction

Orofacial clefts are among the most common congenital anomalies of the head and neck, with a global prevalence of approximately one in 700 live births and marked geographic and ethnic variation [1,2]. Although often grouped together, cleft lip with or without cleft palate and cleft palate alone are developmentally distinct entities. Clefting of the lip results from failure of fusion between the maxillary and medial nasal prominences between the fourth and seventh gestational weeks, whereas cleft palate arises from disturbed elevation, growth, or fusion of the palatal shelves between the seventh and twelfth weeks [3]. The molecular programmes governing the two events overlap only partly: in mice, conditional inactivation of Bmp4 in the facial primordia produces isolated cleft lip, and the labial and palatal defects of Bmp receptor mutants arise through different cellular mechanisms—increased apoptosis in the medial nasal process in the first case, and reduced mesenchymal proliferation in the maxillary process in the second [4,5]. This embryological separation is reflected in differing genetic architectures [6,7] and, as the present study shows, in differing early clinical trajectories.

Prenatal ultrasound has become a routine component of antenatal care, and detection of orofacial clefts is an established secondary aim of the mid-trimester anomaly scan. However, detection performance is highly uneven. A systematic review of 27 studies of second-trimester transabdominal ultrasound in unselected low-risk populations found wide variation: detection ranged from 9% to 100% for cleft lip with or without cleft palate, but only from 0% to 22% for cleft palate only [8]. More recent syntheses confirm that neither two- nor three-dimensional ultrasound achieves reliable visualisation of the secondary palate in routine practice [9,10]. Advances in targeted foetal imaging, including dedicated multiplanar palatal views and axial transverse approaches to the hard palate, have shown potential to improve visualisation in selected or high-risk pregnancies [11,12,13,14]. These techniques, however, require additional expertise, favourable imaging conditions, or second-line assessment, and their reported accuracy may not translate directly to routine population screening. Isolated cleft palate therefore remains a major diagnostic blind spot in standard antenatal care. Population-based series illustrate both the scale of the problem and the difficulty of studying it: a Norwegian prospective study covering 49,314 deliveries in an unselected population identified only 101 foetuses or newborns with facial clefts over 18 years and was among the first to examine whether detection improved across successive periods [15]. Cohorts assembled from routine antenatal care therefore accumulate slowly, and single-country series remain the principal source of evidence on how screening performs in practice rather than in specialist settings. The anatomical explanation is straightforward: the lip is displayed on the coronal facial view included in standard protocols, whereas the secondary palate lies behind the alveolar ridge and requires dedicated planes, favourable foetal position, and considerable operator experience [16,17,18].

Prenatal identification carries documented benefits. It permits structured counselling by the cleft team, planning of delivery in an appropriately resourced unit, early feeding advice, and organisation of multidisciplinary care from the first day of life. Systematic review evidence indicates that prenatal diagnosis followed by planned counselling improves parental preparedness and reduces the psychological impact of the diagnosis [19], and cohort data suggest that prenatal consultation shortens time to definitive care [20]. Conversely, cleft palate is frequently missed not only before birth but also afterward. A United Kingdom audit of 344 infants with cleft palate found that detection was delayed beyond the first day of life in 28% overall and in 37% of those with isolated cleft palate, and that feeding problems and nasal regurgitation were significantly more common in the delayed group [21]. In a Dutch series, the mean age at diagnosis of isolated cleft palate was one year and four months, with a quarter of children diagnosed after their first birthday [22]. The postnatal safety net is therefore weakest for exactly the phenotype that the antenatal one misses.

Feeding difficulty is the earliest functional consequence of clefting and follows a distinct distribution. An intact palate is required to generate the negative intraoral pressure necessary for effective sucking, so infants with palatal involvement cannot feed normally at the breast and commonly require specialised bottles or, in a subset, enteral support [23,24,25]. Difficulty is concentrated in cases of cleft palate only [26,27] and is further aggravated when micrognathia and glossoptosis coexist, as in the Pierre Robin sequence, where airway compromise competes with feeding [28,29].

These two bodies of literature have developed in parallel but have not been integrated. To our knowledge, no previous study has specifically examined whether prenatal detection and neonatal feeding support follow opposite, phenotype-specific patterns within the same cohort. If the phenotypes least visible before birth are those most dependent on early support after birth, the implication for service organisation is direct: relying on prenatal identification to trigger preparation would systematically fail the children who need it most.

We therefore analysed a nationally ascertained referral-centre cohort of children with an orofacial cleft and no clinically recognised syndrome, managed at the single national referral centre in Croatia. Our aims were to quantify prenatal ultrasound detection and neonatal feeding support needs by cleft phenotype, identify factors independently associated with each, and determine the proportion of children who arrived both undiagnosed and requiring assisted feeding.

## 2. Materials and Methods

### 2.1. Study Design

**Figure 1:**
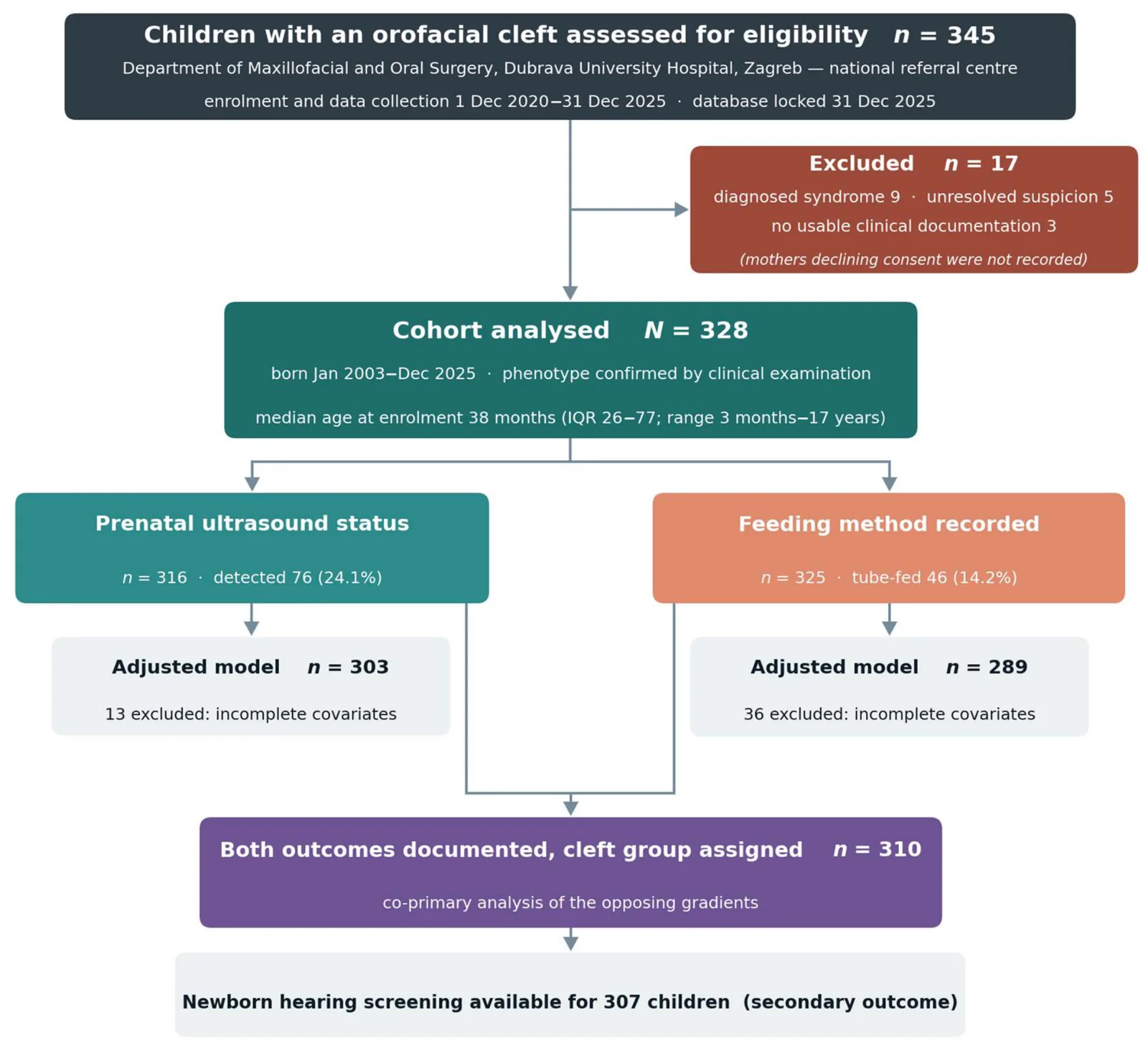
Study flow and cohort composition. Of 345 children assessed for eligibility, 17 were excluded (9 with a diagnosed syndrome, 5 with an unresolved suspicion of a syndrome, 3 without usable clinical documentation); mothers who declined consent were not counted. The analysed cohort comprised 328 children born January 2003–December 2025 (median age at enrolment: 38 months, IQR: 26–77). Documented prenatal ultrasound status was available for 316 children and feeding method for 325; 310 had both outcomes and an assigned cleft group, and the adjusted models included 303 (detection) and 289 (tube feeding) children. Newborn hearing screening (secondary outcome) was available for 307.

This was an ambispective observational cohort study reported in accordance with the STROBE statement for observational studies; the completed checklist is provided as Supplementary Material (Table S1) [30]. The cohort was established prospectively according to a predefined protocol: consecutive eligible children attending the centre were enrolled at the time of a clinical visit, and data were recorded at enrolment using a standardised instrument. Children were enrolled between 1 December 2020 and 31 December 2025, and the database was locked on 31 December 2025. The outcomes analysed here—prenatal ultrasound diagnosis and neonatal feeding method—relate to events that had already occurred and were abstracted retrospectively from medical records. Children born earlier in the period were therefore enrolled at an older age when attending for treatment or follow-up, whereas the most recently born children were enrolled during infancy; the median age at enrolment was 38 months (IQR 26–77; range 3 months to 17 years). Documentation was unavailable for a small number of participants; the number of children contributing to each analysis is stated throughout and summarised in Figure 1.

### 2.2. Setting and Participants

The study was conducted at the Department of Maxillofacial and Oral Surgery, Dubrava University Hospital, Zagreb, designated by the Croatian Ministry of Health as the national referral centre for the surgical treatment of craniofacial malformations. Because all surgically managed orofacial clefts in Croatia are referred to this centre, the cohort represents a nationally ascertained clinical sample rather than a single-institution convenience series. The term describes the referral pathway; the study was not designed to estimate population incidence.

Consecutive children with a clinically confirmed cleft of the lip and/or palate who attended the centre for assessment, treatment or follow-up were eligible. Syndromic status was determined by neonatal assessment followed by evaluation by a paediatric geneticist and a maxillofacial surgeon at the time of final assessment, supplemented by genetic testing where clinically indicated; children in whom a syndrome was diagnosed or in whom a suspicion of a syndrome remained unresolved were excluded, as were children whose records were insufficient for reliable phenotyping. We describe the cohort as having no clinically recognised syndrome, in preference to the categorical term “non-syndromic”, because universal genetic testing was not performed; in a parallel targeted-sequencing substudy of children with cleft palate from the same centre, a pathogenic or likely pathogenic variant was identified in a minority of clinically non-syndromic cases, indicating that clinical assessment alone cannot exclude monogenic disease. The Pierre Robin sequence was recorded as a phenotypic feature rather than as a syndromic diagnosis, and children with the sequence were retained; a sensitivity analysis excluding them is reported in the Supplementary Materials (Table S3).

Written informed consent was obtained from the mothers before inclusion. Mothers who declined participation were not enrolled, and the number of those approached but not consenting was not recorded; the resulting potential for selection bias is addressed in the limitations.

### 2.3. Phenotype Classification

Cleft phenotype was classified according to the international cleft classification adopted at the 1967 Rome congress. Rome congress, which remains in routine use at the centre and distinguishes clefts of the primary palate, the secondary palate and both, on the basis of clinical examination and available documentation, with final confirmation by a maxillofacial surgeon. Six categories were distinguished: incomplete and complete cleft lip, unilateral and bilateral cleft lip and palate, and incomplete and complete cleft palate. For principal analyses these were grouped into cleft lip only, cleft lip and palate, and cleft palate only. Laterality was recorded as left, right or bilateral for clefts involving the lip.

A positive family history of clefting was defined as a reported orofacial cleft in the mother, the father, a sibling or any other relative, ascertained by structured maternal interview at enrolment and not verified against external records. Maternal education, recorded on the standardised instrument at enrolment, was dichotomised as higher—completed post-secondary education, comprising a professional college or university qualification—versus secondary school completed or less. Associated congenital anomalies were defined as additional anomalies not forming part of a syndromic entity, confirmed in the medical record; an isolated patent foramen ovale was not counted as an anomaly, being a physiological variant of infancy that closes spontaneously in most children. Where the record specified the anomaly, it was additionally assigned to one or more organ systems (cardiac, urogenital, ear, limb, central nervous system, mandibular).

### 2.4. Outcomes

Prenatal ultrasound examinations were not performed at the study centre but as part of routine antenatal care delivered across the national maternity network, following the customary schedule of examinations recommended in pregnancy, which includes a mid-trimester morphology scan. Neither the gestational age at examination, the equipment used, the imaging protocol, nor operator experience were recorded in the referral documentation available to us.

Prenatal diagnostic status was ascertained by review of the original antenatal ultrasound reports contained in the child’s documentation, supplemented by the maternity discharge summary, the maternal antenatal records and referral documentation. Antenatal care in Croatia is delivered under the national programme of health-care measures, which for normal pregnancy prescribes three ultrasound examinations, at 10–14, 18–22 and 31–34 weeks of gestation, including the second-trimester examination at which foetal morphology is assessed [31]. Children were classified as detected when an orofacial cleft of any type was recorded as identified on antenatal ultrasound; detection therefore denotes prenatal recognition of any cleft and not necessarily correct delineation of the complete postnatal phenotype, and whether the palatal component had been specifically recognised in children with cleft lip and palate was not separately recorded. In both children with cleft palate only who were classified as detected, the original report explicitly described a cleft of the palate. Children were classified as not detected when the reviewed prenatal ultrasound documentation contained no record of a cleft, and as unknown when that documentation was unavailable; the 12 children with unknown status were excluded from the detection denominator, so an absent record was not equated with non-detection. The estimate should accordingly be read as documented prenatal diagnosis among children reaching the referral centre rather than as the sensitivity of population screening.

The two co-primary outcomes were documented prenatal ultrasound diagnosis of the cleft, recorded as a binary variable, and feeding method during the first weeks of life, categorised as exclusive breastfeeding, breastfeeding with bottle, bottle only, or nasogastric tube feeding with or without bottle. Nasogastric tube feeding was defined as documented enteral feeding through a nasogastric tube for nutritional support during the neonatal period, irrespective of concurrent bottle feeding. The decision to place a tube was made by the treating neonatal team according to local clinical judgement rather than a written protocol, and neither the duration of tube feeding nor the specific indication was recorded systematically; this is addressed in the limitations. Both outcomes were abstracted from medical records and did not rely on maternal recall.

Pre-specified secondary outcomes were the results of newborn hearing screening and the composite outcome of arriving without a prenatal diagnosis while requiring tube feeding. Hearing screening was performed within the Croatian national newborn hearing screening programme, which is delivered in all maternity units and uses transient evoked otoacoustic emissions tested bilaterally before discharge, with the result recorded as pass or refer [32]. A refer result at this initial screening, whether unilateral or bilateral, was analysed as a single category (initial refer result); children with a refer result are recalled for repeat testing within the programme, but the outcome of repeat testing and any subsequent automated auditory brainstem response assessment was not available in our records, so an initial refer result is not equivalent to confirmed hearing impairment.

### 2.5. Statistical Analysis

Categorical variables are presented as counts and percentages with exact binomial (Clopper–Pearson) 95% confidence intervals. Continuous variables were assessed for distribution and were summarised as medians with interquartile ranges and compared with the Kruskal–Wallis or Mann–Whitney test. Proportions were compared using the chi-square test, or Fisher’s exact test where any expected frequency was below five.

Factors independently associated with prenatal detection and with tube feeding were identified using multivariable logistic regression estimated by Newton–Raphson iteration, with Wald confidence intervals. Covariates were specified a priori: for detection, isolated cleft palate, associated anomalies, maternal age, maternal education and year of birth; for tube feeding, isolated cleft palate, micrognathia, associated anomalies, birth weight, gestational age and year of birth. Discrimination was quantified by the area under the receiver operating characteristic curve with DeLong confidence intervals, and calibration was assessed by the Hosmer–Lemeshow test. Models were fitted to estimate independent associations rather than to derive prediction rules, so no operating thresholds were defined.

The temporal trend in detection was assessed by logistic regression on individual-level data with year of birth as a continuous covariate, reported both unadjusted and adjusted; predicted probabilities were obtained by marginal standardisation over the observed covariate distribution. Annual detection rates are displayed descriptively but were not used for inference, because several early years contributed few children. Whether the temporal trend differed between cleft phenotypes was tested by adding a year-of-birth-by-phenotype interaction term and comparing models by likelihood ratio test. Because feeding practice may have changed over a study period spanning two decades, year of birth was included as a covariate in the model for tube feeding.

Model stability was assessed by reporting events per variable, variance inflation factors, optimism-corrected discrimination from 500 bootstrap replications, and a Firth penalised regression as a sensitivity analysis. Missing data were described by variable and, for model covariates, additionally by cleft group and birth period (Table S5); the characteristics of children included in and excluded from each adjusted model were compared (Table S6). Primary analyses were based on complete cases. Multiple imputation was considered but not undertaken because missingness was limited (no model covariate exceeded 7.3%), proportionally concentrated in perinatal variables from the earliest birth years, and there were few auxiliary variables to support the missing-at-random assumption; the possible direction of complete-case bias is considered in the limitations. Because the two co-primary outcomes addressed distinct pre-specified clinical questions rather than repeated tests of one hypothesis, no multiplicity adjustment was applied; effect estimates with confidence intervals are emphasised alongside *p* values. Two-sided *p* values below 0.05 were considered statistically significant.

Analyses were performed in MedCalc software (version 12.5.0, MedCalc Software, Ostend, Belgium; https://www.medcalc.org, accessed on 1 July 2026). Logistic regression was estimated by Newton–Raphson iteration; Firth penalised logistic regression was implemented by direct maximisation of the Jeffreys-penalised likelihood with profile-likelihood confidence intervals; receiver operating characteristic curves and DeLong intervals were computed from the same framework. For this revised version, every reported estimate was independently re-implemented and verified in Python (version 3.12.7, Phyton Software Foundation, Wilmington, DE, USA; NumPy and SciPy); the complete analysis scripts and their output logs are provided with the Supplementary Materials.

### 2.6. Ethics

The study was conducted in accordance with the Declaration of Helsinki. The protocol was approved by the Ethics Committee of the University of Zagreb School of Dental Medicine at its XXI regular session on 12 November 2020 (No. 05-PA-30-XXI-10/2020) and at its XIV regular session on 2 December 2025 (URBROJ 251-60-4/119-2, KLASA 003-01/25-05/14), and by the Ethics Committee of Dubrava University Hospital (No. 2020/2409-06). All data were anonymized, and each participant was assigned a study code.

## 3. Results

### 3.1. Cohort Characteristics

During the enrolment period, 345 children were assessed for eligibility and 17 were excluded (9 with a diagnosed syndrome, 5 with an unresolved suspicion of a syndrome and 3 without usable clinical documentation); the number of mothers approached who declined consent was not recorded (Figure 1). The remaining 328 children with an orofacial cleft and no clinically recognised syndrome were analysed. Sex was documented for 326 children, of whom 177 were boys (54.3%) and 149 girls (45.7%). A cleft group could be assigned to 323 children; the remaining five lacked sufficient documentation for reliable classification and were retained in analyses not requiring phenotype. Among those classified, cleft lip and palate was the most frequent phenotype (143; 44.3%), followed by cleft palate only (106; 32.8%) and cleft lip only (74; 22.9%).

Children were born between 2003 and 2025 (median 2021, IQR 2019–2023), with 90.8% born in 2015 or later. Median gestational age at birth was 39 weeks (IQR 38–40), and median birth weight was 3375 g (IQR 3052–3700); 20 children (6.6%) were born before 37 completed weeks, and 19 (6.0%) weighed less than 2500 g. Median maternal age was 30 years (IQR 26–34). Associated congenital anomalies were documented in 95 children (29.0%), micrognathia in 19 (5.8%) and the Pierre Robin sequence in 13 (4.0%), the latter confined entirely to the cleft-palate-only group. Where the affected system was specified, cardiac anomalies were the most frequent (*n* = 37), followed by mandibular (*n* = 19), urogenital (*n* = 18), ear (*n* = 7), central nervous system (*n* = 5) and limb anomalies (*n* = 3). Cohort characteristics by cleft group are shown in Table 1.

### 3.2. Prenatal Ultrasound Detection

**Figure 2:**
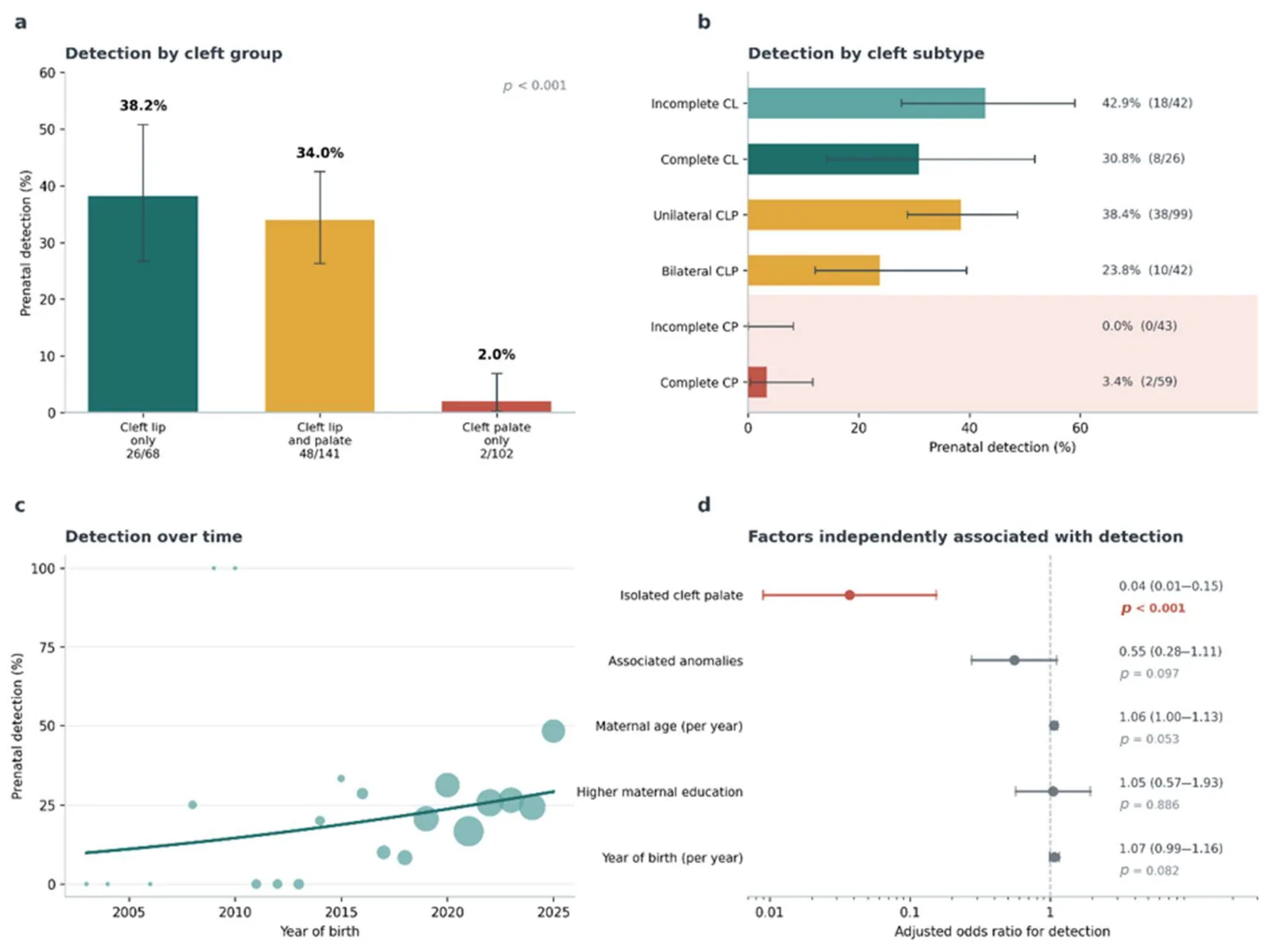
Prenatal ultrasound detection of orofacial clefts. (**a**) Detection by main cleft group with exact 95% confidence intervals; numbers below each bar are detected/assessed. (**b**) Detection by cleft subtype; the shaded band denotes cleft palate only. (**c**) Detection by year of birth, with marker area proportional to the number of children contributing to each year and the solid line showing marginally standardised predicted probabilities from the adjusted model reported in Table 3; annual rates are shown descriptively and no separate trend test is displayed. (**d**) Factors independently associated with prenatal detection from multivariable logistic regression; the estimate significant at *p* < 0.05 is highlighted.

Prenatal ultrasound documentation was available and reviewed for 316 children; in the remaining 12 it was unavailable and diagnostic status was classified as unknown. The cleft was identified before birth in 76 (24.1%; 95% CI 19.4–29.2). Detection differed profoundly by phenotype (*p* < 0.001): 26 of 68 children with cleft lip only (38.2%; 95% CI 26.7–50.8), 48 of 141 with cleft lip and palate (34.0%; 26.3–42.5) and only 2 of 102 with cleft palate only (2.0%; 0.2–6.9) (Figure 2a).

Analysis by six subtypes confirmed the same pattern (*p* < 0.001; Table 2, Figure 2b). The highest rates were observed for incomplete cleft lip and unilateral cleft lip and palate, while the lowest rates were observed for incomplete and complete cleft palate; no child with an incomplete cleft palate was identified before birth. Among clefts involving the lip, detection did not differ by laterality (left 35.2%, right 38.9%, bilateral 31.8%; *p* = 0.765).

In multivariable analysis (*n* = 303; 74 events), isolated cleft palate showed the strongest independent negative association with detection (adjusted OR 0.037; 95% CI 0.009–0.154; *p* < 0.001), corresponding to approximately 96% lower adjusted odds of detection; the reciprocal estimate corresponds to approximately 27-fold higher adjusted odds of non-detection (95% CI 6.5–115.4). No other covariate reached statistical significance; maternal age showed a borderline positive association with detection (adjusted OR 1.061; 95% CI 0.999–1.127; *p* = 0.053). Model discrimination was good (AUC 0.781; 95% CI 0.728–0.835; Nagelkerke R^2^ 0.284; Hosmer–Lemeshow *p* = 0.126), and all variance inflation factors were below 1.16 (Table 3, Figure 2d). Thirteen children with a documented prenatal status were excluded from the adjusted model because of incomplete covariate data; their characteristics are compared to those of included children in Table S6.

Observed detection was higher among children born later in the study period: 4/30 (13.3%) in children born in 2003–2014, 12/66 (18.2%) in 2015–2019 and 60/219 (27.4%) in 2020–2025. In unadjusted logistic regression, the odds of detection increased by 1.095 per year of birth (95% CI 1.012–1.185; *p* = 0.023), but after adjustment for phenotype, associated anomalies, maternal age, and maternal education, the estimate was attenuated and no longer statistically significant (adjusted OR 1.071; 95% CI 0.991–1.157; *p* = 0.082). Marginally standardised predicted detection rose from 11.1% in 2005 to 18.8% in 2015 and 29.2% in 2025. A formal test of interaction between year of birth and cleft phenotype was not significant (likelihood ratio statistic 0.11; *p* = 0.735), so we cannot conclude that any temporal improvement differed between phenotypes, although the absolute number of detected cleft palate only cases remained very small throughout (Figure 2c). Given the small number of children contributing to the earliest birth years, the enrolment of older birth cohorts at later ages, and the attenuation of the estimate after adjustment, this temporal pattern is presented as descriptive and hypothesis-generating.

### 3.3. Neonatal Feeding Support

**Figure 3:**
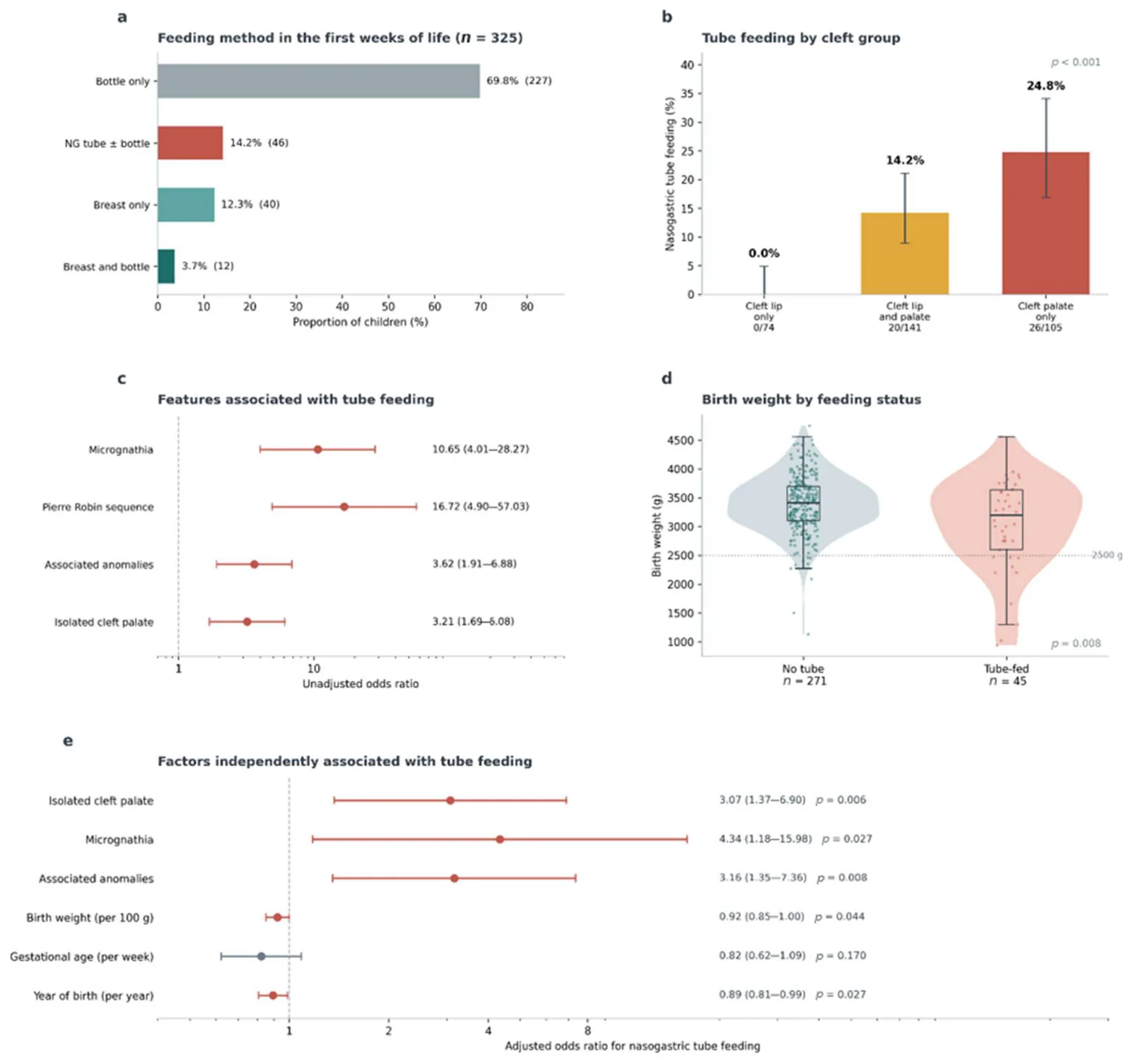
Neonatal feeding support. (**a**) Feeding method during the first weeks of life. (**b**) Requirement for nasogastric tube feeding by main cleft group, with exact 95% confidence intervals. (**c**) Clinical features associated with tube feeding, shown as unadjusted odds ratios on a logarithmic scale. (**d**) Birth weight by feeding status, shown as violin and box plots with individual values; the dotted line marks 2500 g. (**e**) Factors independently associated with tube feeding from multivariable logistic regression; full model specification is given in Table 5.

The feeding method in the first weeks of life was documented for 325 children. Bottle feeding alone was reported in 227 (69.8%), exclusive breastfeeding in 40 (12.3%) and combined breastfeeding with bottle in 12 (3.7%). Nasogastric tube feeding was required by 46 children (14.2%; 95% CI 10.6–18.4) (Figure 3a). The full distribution of feeding categories by cleft group is provided in Table S4.

The requirement for tube feeding followed the opposite gradient to prenatal detection (*p* < 0.001): no child with cleft lip only required a tube (0 of 74), compared with 20 of 141 with cleft lip and palate (14.2%; 95% CI 8.9–21.1) and 26 of 105 with cleft palate only (24.8%; 16.9–34.1) (Figure 3b). Relative to clefts involving the lip, isolated cleft palate carried an odds ratio of 3.21 (95% CI 1.69–6.08) for requiring tube feeding. Analysis by subtype showed that the burden was concentrated in complete cleft palate, in which 23 of 60 children (38.3%; 95% CI 26.1–51.8) required a tube (Table 4).

In univariable analysis, tube feeding was strongly associated with micrognathia (57.9% versus 11.4%; OR 10.65; 95% CI 4.01–28.27; *p* < 0.001), the Pierre Robin sequence (69.2% versus 11.9%; OR 16.72; 4.90–57.03; *p* < 0.001) and the presence of associated anomalies (26.6% versus 9.1%; OR 3.62; 1.91–6.88; *p* < 0.001) (Figure 3c). Median birth weight was lower in tube-fed infants (3200 g, IQR 2600–3640) than in those not requiring a tube (3410 g, IQR 3100–3700; *p* = 0.008) (Figure 3d).

In the multivariable model (*n* = 289; 41 events; 6.8 events per variable), isolated cleft palate, micrognathia, associated anomalies, and birth weight remained independently associated with tube feeding, and the odds of tube feeding declined with later year of birth (Table 5, Figure 3e). Each additional year of birth was associated with an adjusted odds ratio of 0.893 (95% CI 0.808–0.987; *p* = 0.027), corresponding to a decline of approximately 68% in the adjusted odds over a decade. Variance inflation factors were all below 1.34, indicating no material collinearity. Discrimination was good (AUC 0.829; 95% CI 0.757–0.901; optimism-corrected AUC 0.807 after 500 bootstrap replications) with no evidence of miscalibration on the Hosmer–Lemeshow test (*p* = 0.885). Because the number of events per variable was below the conventional threshold of ten, the model was refitted using Firth penalised regression with profile-likelihood confidence intervals as a sensitivity analysis; effect estimates were closely comparable (Table S2, Supplementary Materials). Given the limited number of events, the estimates for the less frequent covariates, par-titularly micrognathia (95% CI 1.18–15.98), are imprecise and should be regarded as exploratory and supportive rather than definitive; interpretation rests on effect sizes and confidence intervals rather than on significance thresholds alone. Thirty-six children with a documented feeding method were excluded from the adjusted model because of incomplete covariate data; their characteristics are compared with those of included children in Table S6.

**Table 5:** Factors independently associated with the requirement for nasogastric tube feeding (multivariable logistic regression analysis).

Predictor	β	Odds Ratio	95% CI	*p*
Isolated cleft palate	1.122	3.07	1.37–6.89	0.007
Micrognathia	1.467	4.34	1.18–15.98	0.028
Associated anomalies	1.150	3.16	1.36–7.36	0.008
Birth weight (per 100 g)	−0.082	0.92	0.85–1.00	0.044
Gestational age (per week)	−0.195	0.82	0.62–1.09	0.170
Year of birth (per year)	−0.113	0.89	0.81–0.99	0.027

*n* = 289; 41 events (6.8 per variable); AUC 0.829 (95% CI 0.757–0.901); optimism-corrected AUC 0.807; Nagelkerke R^2^ 0.313; Hosmer–Lemeshow *p* = 0.885. Birth weight is modelled per 100 g, gestational age per week, and year of birth per year (centred at 2015). All variance inflation factors were below 1.34.

### 3.4. Opposing Gradients Across Phenotypes

**Figure 4:**
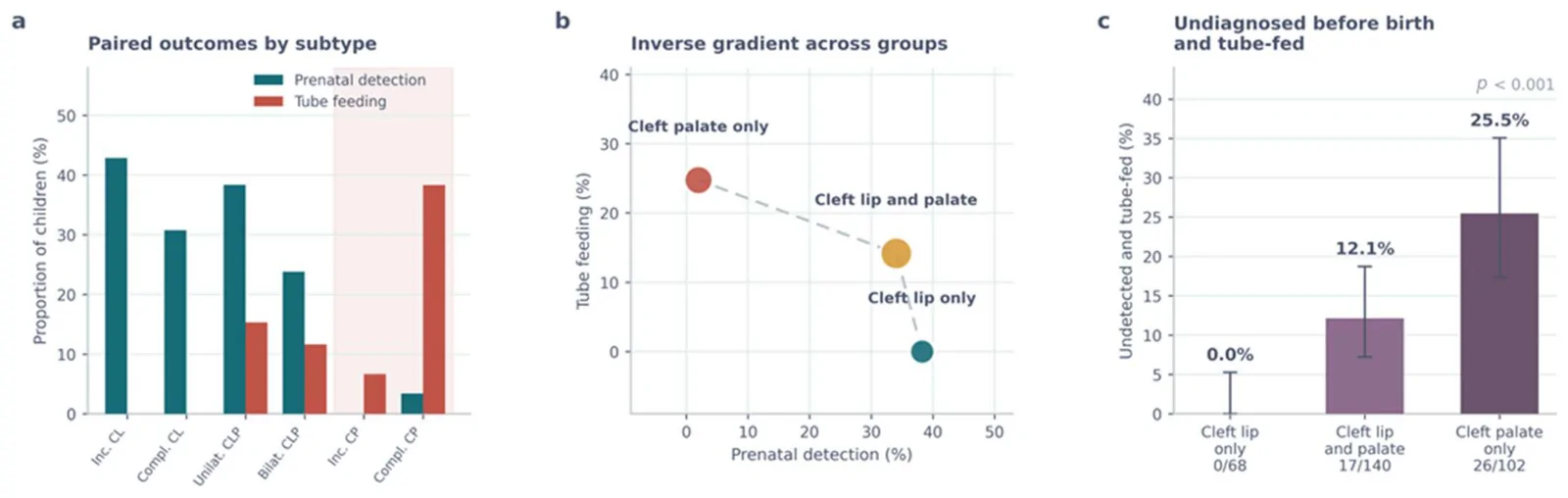
The inverse relationship between prenatal detectability and neonatal feeding support. (**a**) Paired comparison by cleft subtype; the shaded band denotes cleft palate only. (**b**) Monotone inverse gradient across the three main cleft groups, with marker area proportional to group size. (**c**) Proportion of children who arrived without a prenatal diagnosis and subsequently required tube feeding.

Plotting the two outcomes together revealed a monotonic inverse gradient across the three main cleft groups (Figure 4a,b). Cleft lip only combined the highest detection rate (38.2%) with no requirement for tube feeding; cleft lip and palate occupied an intermediate position (34.0% and 14.2%); and cleft palate only combined the lowest detection rate (2.0%) with the highest tube-feeding requirement (24.8%). Because the gradient is based on three groups, no formal correlation coefficient is reported; the relationship rests on two independently significant tests running in opposite directions.

The clinical consequence was quantified directly. Among 310 children with both outcomes documented and an assigned cleft group, 43 (13.9%; 95% CI 10.2–18.2) arrived without a prenatal diagnosis and subsequently required tube feeding. This composite differed strongly by phenotype (*p* < 0.001): 0.0% for cleft lip only, 12.1% for cleft lip and palate, and 25.5% for cleft palate only (Table 6, Figure 4c). Viewed from the opposite direction, 43 of the 46 children who required tube feeding (93.5%; 95% CI 82.1–98.6) had not been identified before birth.

### 3.5. Newborn Hearing Screening

**Figure 5:**
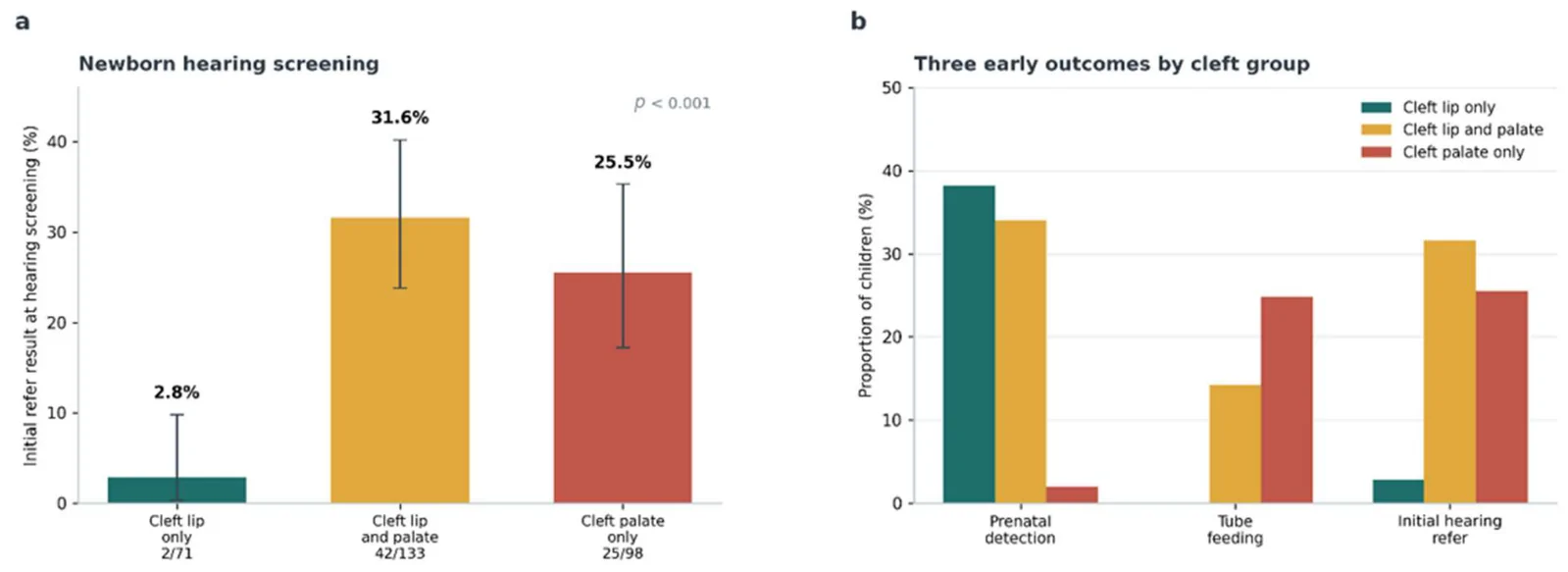
Early clinical burden by cleft group. (**a**) Initial refer result at newborn hearing screening with exact 95% confidence intervals. (**b**) The three outcomes displayed together: prenatal detection falls and tube feeding rises with involvement of the palate, while an initial hearing-screening refer result is elevated in both groups with palatal involvement without a monotonic gradient.

Newborn hearing screening results were available for 307 children, of whom 70 (22.8%; 95% CI 18.2–27.9) had an initial refer result. Phenotype was also known for 302 of these children, and among them the result differed markedly by cleft group (*p* < 0.001): 42 of 133 with cleft lip and palate (31.6%; 95% CI 23.8–40.2), 25 of 98 with cleft palate only (25.5%; 17.2–35.3) and only 2 of 71 with cleft lip only (2.8%; 0.3–9.8). Referral was thus substantially more frequent in both groups with palatal involvement than in children with cleft lip only, although it did not increase monotonically from cleft lip and palate to cleft palate only; this secondary outcome contrasts cleft lip only with clefts involving the palate and provides supportive, rather than independently confirmatory, evidence that early clinical burden is concentrated in palatal clefts (Figure 5). An initial refer result on otoacoustic emission screening is not equivalent to confirmed hearing impairment.

### 3.6. Sensitivity Analyses

Excluding the 13 children with the Pierre Robin sequence, all of whom had isolated cleft palate, did not alter the direction or significance of the principal findings. Detection in the cleft-palate-only group remained at 1 of 89 (1.1%; 95% CI 0.0–6.1), and tube feeding at 17 of 92 (18.5%; 11.1–27.9), and isolated cleft palate remained independently associated with tube feeding in the multivariable model (adjusted OR 2.68; 95% CI 1.16–6.18; *p* = 0.021), although micrognathia no longer reached significance once the sequence was removed (Table S3). Firth penalised regression produced estimates closely comparable with the unpenalised model (Table S2).

## 4. Discussion

In a nationally ascertained referral-centre cohort of 328 children with an orofacial cleft and no clinically recognised syndrome, prenatal detectability and neonatal feeding support requirements followed opposite gradients across cleft phenotypes. Cleft palate only was detected before birth in 2.0% of cases, compared with 38.2% for cleft lip only, yet required nasogastric tube feeding in 24.8% of cases compared with none. Almost all children who required tube feeding (93.5%) had not been identified prenatally. Throughout, nasogastric tube use denotes documented tube placement decided by the treating team’s clinical judgement rather than a standardised measure of feeding severity, so the phenotype-specific differences partly reflect clinical practice in addition to physiological impairment. An initial refer result at newborn hearing screening was likewise concentrated in children with palatal involvement, although without a monotonic gradient across the three groups, indicating that the concentration of early burden in palatal clefts is not confined to feeding.

The two gradients are individually unsurprising; their juxtaposition is the point. Each literature, read alone, generates a coherent but incomplete service implication. The prenatal imaging literature concludes that detection of the secondary palate should be improved; the feeding literature concludes that infants with palatal clefts need early specialist support. Read together, they identify a structural gap that neither reveals on its own: the diagnostic pathway performs best precisely where the early clinical need is lowest, so any system that uses antenatal findings to trigger neonatal preparation is calibrated against the children who need that preparation most.

Our detection figures align closely with the published range. The systematic review by Maarse and colleagues reported detection of isolated cleft palate ranging from zero to 22% in low-risk populations [8], and subsequent syntheses have confirmed that neither two- nor three-dimensional ultrasound reliably visualises the secondary palate in routine screening [9,10]. Our value of 2.0% lies at the lower end of that range, and the complete absence of detected cases among incomplete cleft palates is consistent with the expectation that the least conspicuous defects are the least likely to be seen.

The overall detection rate of 24.1% lies in the lower part of the reported spectrum. Three features of our cohort explain this. First, its composition is unusual: cleft palate only accounted for 32.8% of classified children, a higher share than in many surgical series, and because this phenotype is almost never detected, it depresses the overall figure. Second, ascertainment was from routine antenatal care delivered across a national maternity network rather than from tertiary referral scanning, and detection rates from routine practice are consistently lower than those reported from specialist units. Third, the cohort spans more than two decades of evolving imaging practice. Direct comparison with population-based series is complicated by differences in ascertainment: studies that enumerate every delivery in a defined region capture terminations and stillbirths as well as live births, whereas a surgical referral cohort such as ours necessarily begins after birth and after referral [15]. Our figure should therefore be read as the proportion of children reaching cleft surgery who had been identified antenatally, which is the quantity relevant to service planning, rather than as a screening sensitivity.

Our tube-feeding rate for cleft palate only (24.8%) is broadly consistent with the 32% reported in a Dutch retrospective series of 90 children with cleft palate only [26], and the concentration of feeding difficulty in palatal clefts is well established in both physiological and clinical studies [23,27]. The multisite CORNET study demonstrated that infants with the Pierre Robin sequence experience feeding difficulty more frequently than those with isolated cleft palate (81% versus 61%) and receive all forms of feeding intervention more often [29], which mirrors our finding that micrognathia showed the strongest, though imprecisely estimated, independent association with tube feeding, and that this effect attenuated once children with the sequence were removed.

The absence of tube feeding among 74 children with cleft lip only deserves emphasis. No child in this group received a nasogastric tube, and the group therefore serves as an internal comparison: the phenotype that is most visible antenatally imposed no documented requirement for enteral support. This strengthens the inference that palatal integrity, rather than facial clefting in general, drives the requirement for enteral support.

The explanation for the opposing gradients is anatomical rather than coincidental. Prenatal visualisation depends on a surface defect accessible to the standard coronal facial plane, which the lip provides and the secondary palate does not; the palate lies behind the alveolar ridge and requires dedicated planes, a favourable foetal lie, and considerable operator experience [16,17,18]. Early feeding capacity, by contrast, depends on the integrity of the palate, which permits generation of the negative intraoral pressure required for effective sucking; the lip contributes comparatively little to this mechanism [23,24,25]. The two determinants are therefore governed by different anatomical structures whose involvement varies inversely across the cleft spectrum, and isolated cleft palate maximises the mismatch: invisible on the plane that is routinely obtained, yet functionally decisive for feeding.

This account also offers a plausible reason why general improvement in imaging has not closed the gap. Better equipment and more experienced operators improve visualisation of structures already within the imaging plane; by themselves, they do not bring the secondary palate into it. Closing the gap requires a change in technique rather than a change in resolution, which is precisely what the dedicated palatal approaches described in recent years attempt to provide [11,12,13,14]. Our data are consistent with this interpretation: observed detection rose across the study period, from 13.3% in children born before 2015 to 27.4% in those born from 2020 onwards, yet only two cases of cleft palate only were detected in the entire cohort. We note, however, that the temporal increase was attenuated and no longer statistically significant after adjustment (adjusted OR 1.071 per year; *p* = 0.082), and that the test for a difference in trend between phenotypes was not significant, so this interpretation is offered as an explanation consistent with our data rather than as a demonstration, and the temporal pattern itself should be regarded as descriptive and hypothesis-generating.

An unanticipated finding was that the adjusted odds of tube feeding fell with later year of birth (OR 0.89 per year; 95% CI 0.81–0.99), amounting to approximately 68% lower adjusted odds over a decade, while the phenotype effects persisted. Because the indication for tube placement was not recorded and no written feeding protocol was in place, the reason for this decline cannot be established from our data.

Two interpretations carry different implications. If the decline reflects better support—wider availability of specialised cleft feeding bottles, growing staff experience and more consistent early involvement of the cleft team—then the residual burden is potentially modifiable and the appropriate response is to extend those measures. If, instead, it reflects a gradual rise in the threshold for placing a tube, the underlying feeding difficulty may be unchanged, and the apparent improvement would be an artefact of documentation and practice. The two possibilities cannot be distinguished with categorical feeding data alone; prospective recording of the indication for and duration of enteral support, together with weight trajectory in the first weeks, would separate them.

The randomised evidence base for feeding interventions in this population is limited. A Cochrane review of feeding interventions in infants with clefts identified only five randomised studies, comprising fewer than three hundred infants in total. It found that squeezable bottles appear easier to use than rigid ones, but without demonstrable differences in growth; only weak evidence favouring breastfeeding over spoon-feeding after lip repair; no evidence that maxillary plates assist growth; and, notably, no studies at all evaluating maternal advice or support, the intervention most widely delivered in practice [32]. The decline we observed therefore cannot be attributed to any specific intervention of proven effect, because no such intervention has been established. This is itself an argument for treating our finding as hypothesis-generating and for the trials proposed below.

The most direct implication is that prenatal diagnosis cannot serve as the trigger for neonatal feeding preparation. In our cohort, 93.5% of tube-fed infants arrived without a prenatal diagnosis, and among children with cleft palate only, a quarter arrived both undiagnosed and requiring assisted feeding. Any care pathway in which specialist feeding support is mobilised on the basis of antenatal findings will therefore systematically miss the majority of infants who need it. The corollary is that feeding preparedness must be a property of the maternity unit rather than of the individual pregnancy.

Three practice considerations arise from the observed associations; none was tested as an intervention in this study. First, every newborn with a palatal cleft should undergo structured feeding assessment by appropriately trained staff early in the neonatal period, irrespective of prenatal findings; our data do not identify an optimal time window, and the 24 h threshold commonly used in cleft protocols was not tested here, nor did we evaluate whether structured assessment reduces tube use, weight loss or length of hospital stay. Second, maternity units should have specialised feeding bottles and staff competent in their use, since sucking performance in infants with clefts depends heavily on the feeding method used, and most reported series describe successful oral feeding with adapted equipment [23,27]. Third, documented micrognathia could trigger airway assessment together with intensified feeding surveillance, given that it was the strongest factor independently associated with tube feeding in our model and that airway compromise competes with feeding in the Pierre Robin sequence [28,29]. No airway outcome was measured in this study, so this remains a clinically plausible but untested consideration.

A complementary implication concerns postnatal detection. Cleft palate is missed not only before birth but also frequently after it; the mean age at diagnosis of isolated cleft palate in one series was one year and four months, with a quarter of children diagnosed beyond the first birthday [22]. Systematic inspection and palpation of the palate in every newborn, rather than inspection alone, is therefore warranted, since submucous clefts and clefts of the soft palate are readily overlooked visually. The audit by Habel and colleagues is instructive here: among trainee paediatricians in the referring units, few recalled having received any specific instruction on examination of the palate, and narrow V-shaped and soft palate clefts were the ones most likely to be missed [21]. The barrier is thus not principally one of equipment or time but of training and of a standard technique consistently applied, which is a more tractable target than improving foetal imaging. Our hearing screening data reinforce the same message from a third direction: initial refer rates were an order of magnitude higher in children with palatal involvement than in those with cleft lip only, so the newborn period is when the burden of palatal clefting first becomes measurable across several domains simultaneously.

The principal strength is national clinical ascertainment through a single referral pathway, which reduces the referral bias affecting single-centre series in decentralised systems. Both co-primary outcomes were abstracted from medical records rather than obtained by maternal report, so neither depends on recall. Phenotype was verified by clinical examination with final confirmation by a maxillofacial surgeon. A third outcome provided supportive secondary evidence that early clinical burden is concentrated in palatal clefts, and the principal findings were robust to exclusion of children with the Pierre Robin sequence and to penalised estimation.

Several limitations require acknowledgement. Prenatal ultrasound was performed outside the study centre as part of routine antenatal care; gestational age at examination, equipment, and operator experience were not documented in the available records, so we cannot determine to what extent the low detection of cleft palate reflects the intrinsic limits of the standard views rather than variation in practice. In addition, whether the palatal component had been recognised prenatally in children with cleft lip and palate was not separately recorded, and although prenatal ultrasound documentation was reviewed for every child in the detection denominator, the number and timing of examinations in each individual pregnancy could not be reconstructed; the estimates therefore describe documented prenatal diagnosis among children reaching the referral centre rather than screening sensitivity. Enrolment required maternal consent at a clinical visit, and mothers who declined were not enrolled; because refusals were not recorded, the extent of any resulting selection bias cannot be quantified. Since enrolment took place at a clinical visit rather than at birth, children who died before referral, whose pregnancies were terminated after prenatal diagnosis, or who were never referred are not represented, which is likely to under-represent the most severe presentations and may also under-represent prenatally detected cases, biassing our detection estimate downwards. Ascertainment covers surgically managed clefts and therefore describes the treated population rather than population incidence.

The cohort spans more than two decades during which practice changed, which we addressed by modelling the temporal trend but cannot fully resolve; the unadjusted and adjusted estimates of that trend differed, and we have reported both rather than emphasising the more favourable one. Feeding method was recorded categorically without duration of tube feeding, administered volumes, or the specific indication for placement, and the decision to place a tube reflected clinical judgement rather than a written protocol, so the outcome combines physiological need with local practice; it is accordingly reported as documented nasogastric tube use rather than as a comprehensive measure of feeding difficulty. The number of events per variable in the tube-feeding model was below the conventional threshold of ten, which we addressed by a Firth-penalised estimation and bootstrap optimism correction. These methods address small-sample estimation and overfitting but not selection related to complete-case analysis: the adjusted models excluded 13 children with a documented prenatal status and 36 with a documented feeding method because of incomplete covariates; their characteristics are compared with included children in Table S6, and although the near-identical outcome and phenotype distributions make substantial bias unlikely, its direction cannot be established with certainty. Family history was ascertained by maternal report without external verification. Sex could not be determined in 2 children and the cleft group in 5, and outcome data were incomplete for a further small number, so denominators differ between analyses. Newborn hearing screening was analysed only as the initial otoacoustic emission refer result; repeat testing and auditory brainstem-response outcomes were unavailable, so referral proportions overestimate confirmed hearing impairment and this outcome remains secondary. Finally, although the national referral pathway supports generalisability within Croatia, prenatal screening protocols differ internationally and detection rates should be compared with that caveat.

Prospective registration of prenatal scanning parameters alongside neonatal outcomes would permit direct assessment of which technical factors improve palatal visualisation. Evaluation of targeted second-line imaging, including dedicated palatal views in selected pregnancies, should weigh incremental detection against cost and workload [9,11]. From the neonatal side, trials of protocolised feeding assessment in all infants with palatal clefts could establish whether earlier structured support reduces the need for enteral feeding, the duration of hospital stay, and the incidence of failure to thrive. Given that no randomised evidence exists on maternal feeding advice and support despite its universal use [33], such a trial would address a genuine gap rather than duplicate existing work, and the outcome we report here—the proportion arriving undiagnosed and requiring enteral support—offers a concrete and easily ascertained endpoint. Croatia has no national cleft registry, and the most recent national epidemiological description of orofacial clefts in the country covers births only up to 1998 [34]. Establishing such a registry and linking it to these data would enable evaluation of whether shortening time to diagnosis alters longer-term speech and hearing outcomes.

## 5. Conclusions

Prenatal ultrasound detectability and neonatal nasogastric tube use follow opposite gradients across orofacial cleft phenotypes. Children with cleft palate only are simultaneously the least likely to be identified before birth and the most likely to require assisted feeding, and 93.5% of all tube-fed infants in this nationally ascertained cohort arrived without a prenatal diagnosis. Prenatal identification cannot therefore be relied upon to trigger neonatal preparation. Our findings support, as practice considerations, routine structured feeding assessment for every newborn with a palatal cleft regardless of antenatal findings, and suggest that documented micrognathia may warrant concurrent airway evaluation. Neither measure was directly evaluated as an intervention in this study. The effectiveness and optimal timing of such assessment were not examined here and remain to be tested prospectively, in line with the trials of protocolised feeding assessment proposed above.

## Figures and Tables

**Table 1:** Cohort characteristics, overall and by main cleft group.

Characteristic	All Children	Cleft Lip Only	Cleft Lip and Palate	Cleft Palate Only	*p*
Children, *n*	328	74	143	106	
Male sex, *n* (%)	177 (54.3)	42 (56.8)	87 (60.8)	45 (43.3)	0.021
Gestational age, weeks—median (IQR)	39 (38–40)	39 (38–40)	39 (38–40)	39 (38–40)	0.778
Preterm birth <37 weeks, *n* (%)	20 (6.6)	1 (1.5)	11 (8.1)	8 (8.2)	0.171
Birth weight, g—median (IQR)	3375 (3052–3700)	3430 (2915–3772)	3290 (3055–3635)	3450 (3062–3700)	0.471
Birth weight <2500 g, *n* (%)	19 (6.0)	3 (4.2)	10 (7.2)	6 (5.9)	0.680
Maternal age, years—median (IQR)	30 (26–34)	30 (26–34)	30 (26–34)	31 (28–34)	0.282
Higher maternal education, *n* (%)	155 (48.3)	34 (46.6)	65 (46.4)	55 (52.9)	0.563
Year of birth—median (IQR)	2021 (2019–2023)	2022 (2021–2024)	2021 (2019–2023)	2021 (2019–2022)	<0.001
Associated anomalies, *n* (%)	95 (29.0)	14 (18.9)	42 (29.4)	38 (35.8)	0.048
Micrognathia, *n* (%)	19 (5.8)	0 (0.0)	2 (1.4)	17 (16.0)	<0.001
Pierre Robin sequence, *n* (%)	13 (4.0)	0 (0.0)	0 (0.0)	13 (12.3)	<0.001
Positive family history, *n* (%)	78 (23.8)	15 (20.3)	36 (25.2)	25 (23.6)	0.722

Denominators vary because of missing data: sex was unknown in 2 children, cleft group in 5, gestational age in 24, birth weight in 10, maternal age in 4, maternal education in 7 and year of birth in 2. Percentages are calculated based on children for whom the variable is available. *p* values are from the chi-square test or Fisher’s exact test for categorical variables and the Kruskal–Wallis test for continuous variables, comparing the three main cleft groups. IQR, interquartile range.

**Table 2:** Prenatal ultrasound detection of orofacial clefts by cleft subtypes.

Cleft Subtype	*N*	*Detected*, n	Detection Rate (%)	95% CI (%)
Incomplete cleft lip	42	18	42.9	27.7–59.0
Complete cleft lip	26	8	30.8	14.3–51.8
Unilateral cleft lip and palate	99	38	38.4	28.8–48.7
Bilateral cleft lip and palate	42	10	23.8	12.1–39.5
Incomplete cleft palate	43	0	0.0	0.0–8.2
Complete cleft palate	59	2	3.4	0.4–11.7

The denominator includes only children with documented prenatal ultrasound status and an assigned subtype (*n* = 311). Chi-square test across subtypes, *p* < 0.001. CI, confidence interval (Clopper–Pearson).

**Table 3:** Factors independently associated with documented prenatal ultrasound detection (multivariable logistic regression analysis).

Predictor	β	Odds Ratio	95% CI	*p*
Isolated cleft palate	−3.310	0.037	0.009–0.154	<0.001
Associated anomalies	−0.592	0.553	0.275–1.112	0.097
Maternal age (per year)	0.059	1.061	0.999–1.127	0.053
Higher maternal education	0.045	1.046	0.567–1.928	0.886
Year of birth (per year)	0.068	1.071	0.991–1.157	0.082

*n* = 303; 74 events (14.8 per variable); AUC 0.781 (95% CI 0.728–0.835); Nagelkerke R^2^ 0.284; Hosmer–Lemeshow *p* = 0.126. Year of birth is centred at 2015. All variance inflation factors were below 1.16.

**Table 4:** Requirements for nasogastric tube feeding by cleft subtype.

Cleft Subtype	N	Tube-Fed, *n*	Proportion (%)	95% CI (%)
Incomplete cleft lip	44	0	0.0	0.0–8.0
Complete cleft lip	30	0	0.0	0.0–11.6
Unilateral cleft lip and palate	98	15	15.3	8.8–24.0
Bilateral cleft lip and palate	43	5	11.6	3.9–25.1
Incomplete cleft palate	45	3	6.7	1.4–18.3
Complete cleft palate	60	23	38.3	26.1–51.8

Denominator includes only children with a documented feeding method and an assigned subtype (*n* = 320). Chi-square test across subtypes, *p* < 0.001.

**Table 6:** Children who arrived without a prenatal diagnosis and subsequently required nasogastric tube feeding.

Cleft Group	N	Undetected and Tube-Fed, *n*	Proportion (%)	95% CI (%)
Cleft lip only	68	0	0.0	0.0–5.3
Cleft lip and palate	140	17	12.1	7.2–18.7
Cleft palate only	102	26	25.5	17.4–35.1

Restricted to children with both prenatal ultrasound status and feeding method documented (*n* = 310). Chi-square test, *p* <0.001.

## Data Availability

All data generated or analysed during this study are included in the published article.

## Supplementary Materials

The following supporting information can be downloaded at https://www.mdpi.com/article/10.3390/children13091124/s1, Table S1. STROBE checklist for cohort studies; Table S2. Firth penalised logistic regression for the requirement for nasogastric tube feeding (sensitivity analysis); Table S3. Sensitivity analysis excluding children with the Pierre Robin sequence; Table S4. Feeding method during the first weeks of life by main cleft group; Table S5. Missing data for model covariates, overall and by cleft group and birth period; Table S6. Characteristics of children included in and excluded from the adjusted models.

## Abbreviations

The following abbreviations are used in this manuscript:

CL	Cleft Lip
CLP	Cleft Lip and Palate
CP	Cleft Palate
NG	Nasogastric
OAE	Otoacoustic Emissions
PRS	Pierre Robin Sequence
