# Supplementary material for: Prenatal Ultrasound Detection and Neonatal Nasogastric Tube Feeding Follow Opposite Gradients Across Orofacial Cleft Phenotypes: A Nationally Ascertained Referral-Centre Cohort Study
Source: Children (Basel). 2026 Aug 22;13(9):1124. doi: 10.3390/children13091124 (PMC13605278; doi:10.3390/children13091124)
Supplement: Supplementary file 1 [file children-13-01124-s001.zip › children-4495814-supplementary.pdf]

Supplementary Materials

Table S1. STROBE checklist for cohort studies

Reporting of this study follows the STROBE recommendations for observational research. The section of the manuscript addressing each item is indicated below; page numbers should be added by the authors after final typesetting.

No.	Item	Recommendation	Reported in
1	Title and abstract	(a) Study design indicated in the title and abstract; (b) structured abstract summarising design, methods, results and conclusions	Title; Abstract
2	Background/rationale	Scientific background and rationale explained	Introduction, paragraphs 1–5
3	Objectives	Specific objectives stated	Introduction, final paragraph
4	Study design	Key elements of design presented early in the paper	2.1. Study Design
5	Setting	Setting, locations and relevant dates, including periods of recruitment and data collection	2.1; 2.2
6	Participants	(a) Eligibility criteria, sources and methods of selection; (b) not applicable (no matching)	2.2. Setting and Participants
7	Variables	Outcomes, exposures, predictors and potential confounders clearly defined	2.3; 2.4
8	Data sources/measurement	Sources of data and details of methods of assessment for each variable	2.3; 2.4
9	Bias	Efforts to address potential sources of bias described	2.2 (consent); 2.5 (adjustment); 4.6 (limitations)
10	Study size	Explanation of how the study size was arrived at	2.2 (consecutive enrolment, national referral pathway)
11	Quantitative variables	Handling of quantitative variables and grouping explained	2.5. Statistical Analysis
12	Statistical methods	(a) All methods including confounding control; (b) subgroup analyses; (c) handling of missing data; (d) not applicable; (e) sensitivity analyses	2.5; 3.6; Tables S2–S6
13	Participants	Numbers at each stage, reasons for non-participation, flow diagram	Figure 1; 3.1
14	Descriptive data	(a) Characteristics of participants and information on exposures and potential confounders; (b) missing data for each variable	Table 1; Table S5
15	Outcome data	Numbers of outcome events or summary measures	3.2; 3.3; Tables 2, 4, 6
16	Main results	(a) Unadjusted and adjusted estimates with confidence intervals; (b) category boundaries reported; (c) not applicable	3.2; 3.3; Tables 2–6
17	Other analyses	Other analyses performed, including subgroups, interactions and sensitivity analyses	3.2 (interaction test); 3.6; Tables S2, S3, S5, S6
18	Key results	Key results summarised with reference to study objectives	4.1. Principal Findings
19	Limitations	Limitations discussed, including sources of potential bias or imprecision	4.6. Strengths and Limitations
20	Interpretation	Cautious overall interpretation considering objectives, limitations, multiplicity and other evidence	4.2–4.5; 5. Conclusions
21	Generalisability	External validity of the study results discussed	4.6 (ascertainment); 4.2 (comparison with published evidence)

No.	Item	Recommendation	Reported in
22	Funding	Source of funding and role of funders	Funding statement

STROBE, Strengthening the Reporting of Observational Studies in Epidemiology. Items 6(b), 12(d) and 16(c) are not applicable to this design.

Table S2. Firth penalised logistic regression for the requirement for nasogastric tube feeding (sensitivity analysis)

Predictor	Odds ratio	95% CI	p
Isolated cleft palate	2.97	1.36–6.59	0.007
Micrognathia	3.99	1.17–14.64	0.027
Associated anomalies	3.04	1.33–6.98	0.009
Birth weight (per 100 g)	0.93	0.85–1.00	0.045
Gestational age (per week)	0.83	0.63–1.09	0.174
Year of birth (per year)	0.90	0.81–0.99	0.029

n = 289; 41 events. Firth penalised maximum likelihood was used because the number of events per variable (6.8) was below the conventional threshold of ten. Confidence intervals are profile-likelihood based and p values are from penalised likelihood-ratio tests. Birth weight is modelled per 100 g, gestational age per week and year of birth per year (centred at 2015). Estimates are closely comparable with the unpenalised model reported in Table 5 of the main text, indicating that small-sample bias did not materially affect the conclusions.

Table S3. Sensitivity analysis excluding children with Pierre Robin sequence

(a) **Co-primary outcomes by cleft group**

Cleft group	Prenatal detection, n/N (%)	Tube feeding, n/N (%)
Cleft lip only	26/68 (38.2)	0/74 (0.0)
Cleft lip and palate	48/141 (34.0)	20/141 (14.2)
Cleft palate only	1/89 (1.1)	17/92 (18.5)

(b) **Independent predictors of tube feeding after exclusion**

Predictor	Odds ratio	95% CI	p
Isolated cleft palate	2.68	1.16–6.18	0.021
Micrognathia	2.59	0.51–13.09	0.249
Associated anomalies	3.41	1.44–8.07	0.005
Birth weight (per 100 g)	0.91	0.84–0.99	0.032
Gestational age (per week)	0.88	0.65–1.19	0.404
Year of birth (per year)	0.88	0.79–0.97	0.011

Thirteen children with Pierre Robin sequence, all of whom had isolated cleft palate, were excluded; n = 279 with 33 events. The direction and significance of the principal findings are unchanged. Micrognathia no longer reaches significance because the sequence accounts for most children with documented micrognathia in this cohort, so the two variables are largely collinear once the sequence is removed.

Table S4. Feeding method during the first weeks of life by main cleft group.

Feeding method	Cleft lip only (n = 74)	Cleft lip and palate (n = 141)	Cleft palate only (n = 105)
Breast only	36 (48.6)	1 (0.7)	3 (2.9)
Breast and bottle	10 (13.5)	0 (0.0)	2 (1.9)
Bottle only	28 (37.8)	120 (85.1)	74 (70.5)
Nasogastric tube ± bottle	0 (0.0)	20 (14.2)	26 (24.8)

Values are n (column %). Restricted to children with a documented feeding method and an assigned cleft group (n = 320); five further children with a documented feeding method but no assigned cleft group were all fed by bottle only. Chi-square test across feeding categories and cleft groups: $\chi^2 = 160.1$, $df = 6$, $p < 0.001$; two of twelve cells had expected frequencies below five. All five breastfed children with cleft palate only (three exclusively breastfed, two breast and bottle) had an incomplete cleft palate. Nasogastric tube ± bottle denotes documented nasogastric tube feeding with or without concurrent bottle feeding.

Table S5. Missing data for model covariates, overall and by cleft group and birth period.

Covariate	Missing, n (%)	CL	CLP	CP	2003–2014 (n = 30)	2015–2019 (n = 71)	2020–2025 (n = 225)
Cleft group (isolated cleft palate)	5 (1.5)	—	—	—	1	0	4
Micrognathia	0 (0.0)	0	0	0	0	0	0
Associated anomalies	0 (0.0)	0	0	0	0	0	0
Maternal age	4 (1.2)	1	1	2	0	1	1 *
Higher maternal education	7 (2.1)	1	3	2	0	3	4
Birth weight	10 (3.0)	2	4	4	3	3	3 *
Gestational age	24 (7.3)	9	7	8	6	5	13
Year of birth	2 (0.6)	0	0	2	—	—	— *

*Percentages are of the full cohort (N = 328). CL, cleft lip only; CLP, cleft lip and palate; CP, cleft palate only. Counts by cleft group exclude the five children without an assigned group; counts by birth period exclude the two children without a recorded year of birth (rows marked *: two children missing maternal age and year of birth, and one child missing birth weight, had no recorded year of birth). Relative to the size of each birth cohort, missing gestational age was most frequent among children born 2003–2014 (6/30, 20.0%) compared with 2015–2019 (5/71, 7.0%) and 2020–2025 (13/225, 5.8%), consistent with less complete perinatal documentation in older records.*

Table S6. Characteristics of children included in and excluded from the adjusted models (complete-case analysis).

(a) Model for documented prenatal detection

Characteristic	Included (n = 303)	Excluded (n = 13)	p
Detected prenatally, n (%)	74 (24.4)	2/13 (15.4)	0.741
Cleft palate only, n (%)	99 (32.7)	3/8 (37.5)	0.720
Associated anomalies, n (%)	89 (29.4)	3/13 (23.1)	0.763

Higher maternal education, n (%)	148 (48.8)	2/6 (33.3)	0.685
Maternal age, years — median (IQR)	30 (26–34)	28 (22–35)	0.655
Year of birth — median (IQR)	2021 (2019–2023)	2020 (2019–2024)	0.931

(b) Model for nasogastric tube feeding

Characteristic	Included (n = 289)	Excluded (n = 36)	p
Tube-fed, n (%)	41 (14.2)	5/36 (13.9)	1.000
Cleft palate only, n (%)	94 (32.5)	11/31 (35.5)	0.841
Micrognathia, n (%)	16 (5.5)	3/36 (8.3)	0.453
Associated anomalies, n (%)	85 (29.4)	9/36 (25.0)	0.698
Birth weight, g — median (IQR)	3400 (3070–3700)	3250 (2600–3650)	0.309
Gestational age, weeks — median (IQR)	39 (38–40)	38 (38–39)	0.060
Year of birth — median (IQR)	2021 (2019–2023)	2020 (2015–2022)	0.017

Children were excluded from each model solely because of incomplete covariate data. Denominators for excluded children vary because the compared characteristic was itself sometimes the missing variable; values are calculated on children with the variable available. p values are from Fisher's exact test for categorical variables and the Mann–Whitney test for continuous variables. Among children excluded from the tube-feeding model, maternal age (median 28 versus 30 years; p = 0.010) and higher maternal education (25.8% versus 50.5%; p = 0.013) also differed from included children; neither variable is a covariate of that model. The proportion tube-fed was virtually identical in included and excluded children (14.2% versus 13.9%), and the five tube-fed children excluded account for the difference between the 46 documented events and the 41 events modelled.
